# Supplementary material for: Mining the Genome of Bacillus velezensis VB7 (CP047587) for MAMP Genes and Non-Ribosomal Peptide Synthetase Gene Clusters Conferring Antiviral and Antifungal Activity
Source: Microorganisms. 2021 Dec 3;9(12):2511. doi: 10.3390/microorganisms9122511 (PMC8708206; doi:10.3390/microorganisms9122511)
Supplement: Supplementary file 1 [file microorganisms-09-02511-s001.zip › microorganisms-1445617 - supplementary .pdf]

Figure S1 Phylogenetic tree representing the evolutionary relationships among the strains of *Bacillus velezensis* in comparison with test isolate VB7.

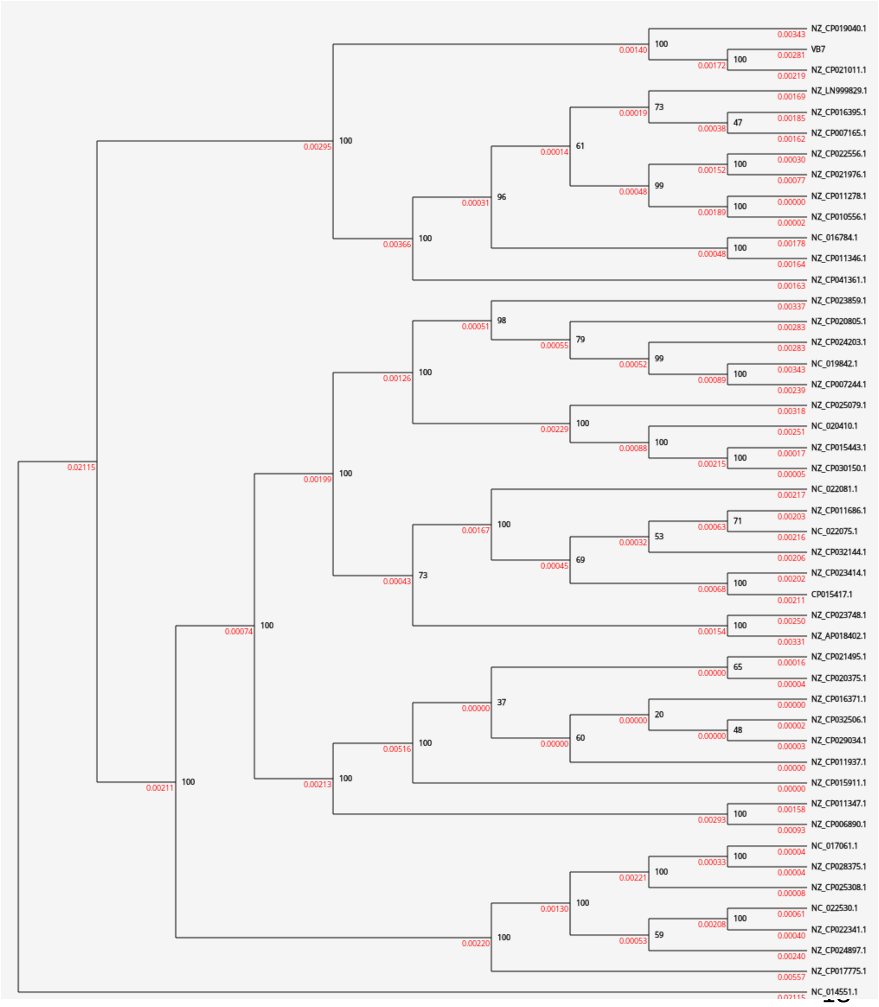

Figure S2 Violin plot representing the dispersion of the identified ORFs count per genome.

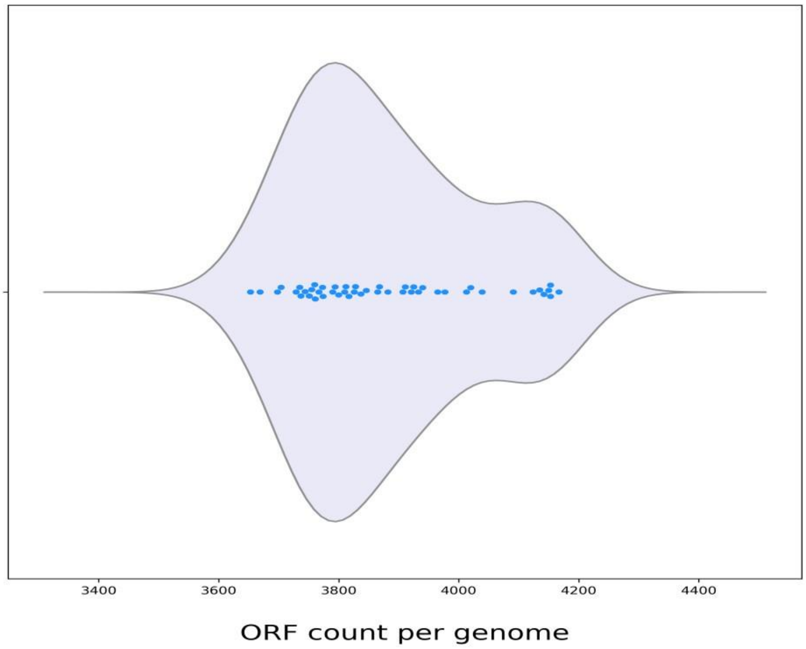

Fig S3. Region-1 coding for secondary metabolite, NRPS in genome of *B. velezensis*

VB7

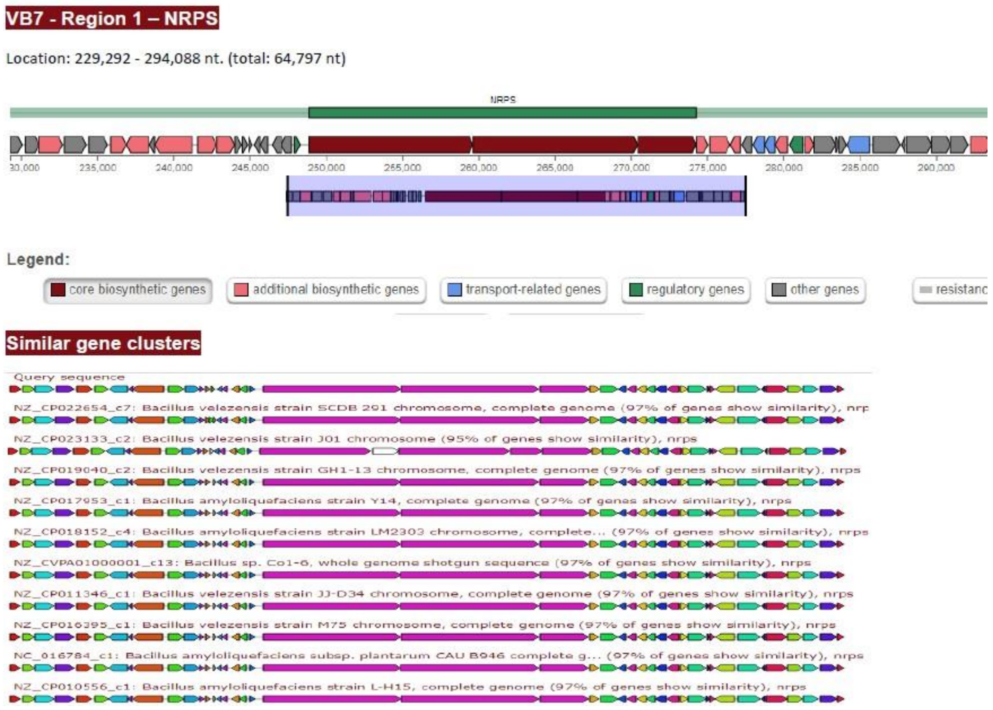

Figure S4. Region-2 coding for secondary metabolite, PKS like in genome of *Bacillus velezensis* – VB7

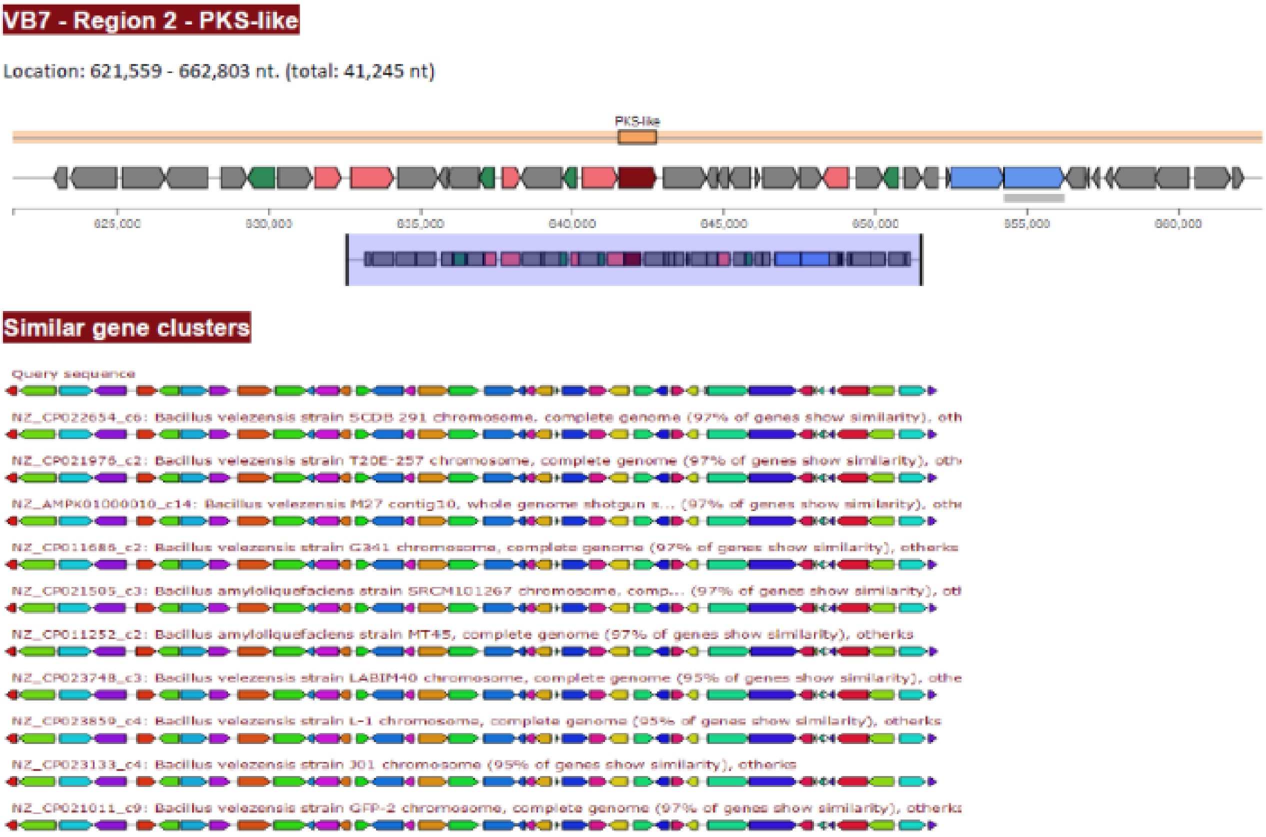

Figure S5 Region-3 coding for secondary metabolite, terpene in genome of *Bacillus velezensis* – VB7

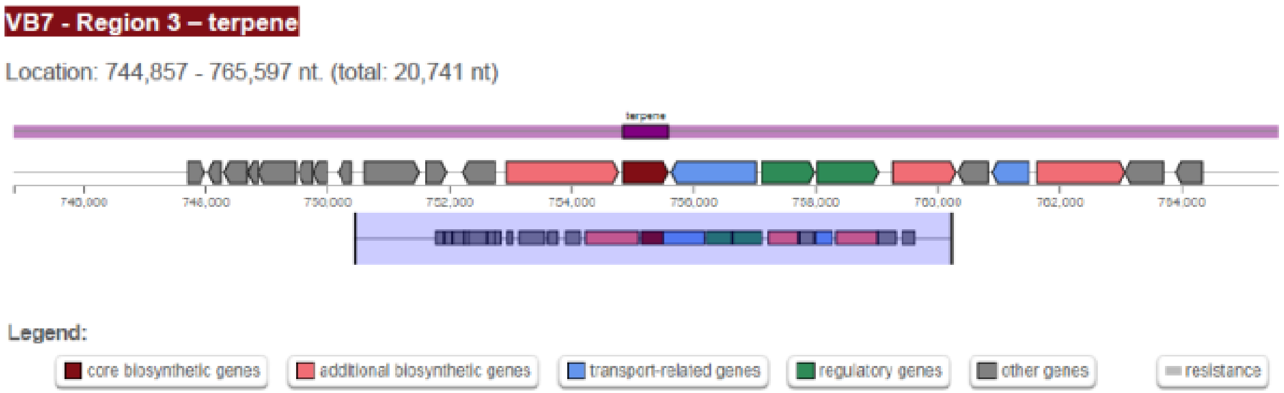

Figure S6 mRegion-4 coding for secondary metabolite, NRPS, betalactone, transAT-PKS in genome of *Bacillus velezensis* – VB7

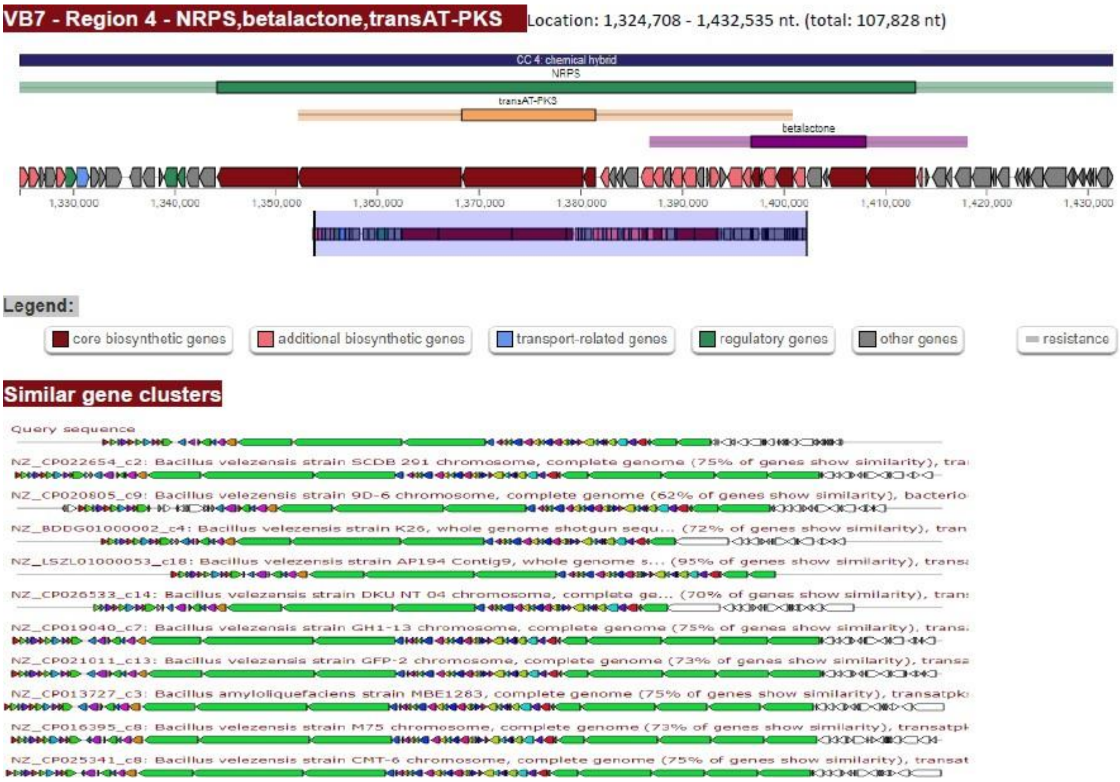

Figure S7 Region-5 coding for secondary metabolite, transAT-PKS, transAT-PKS-like in genome of *Bacillus velezensis* – VB7

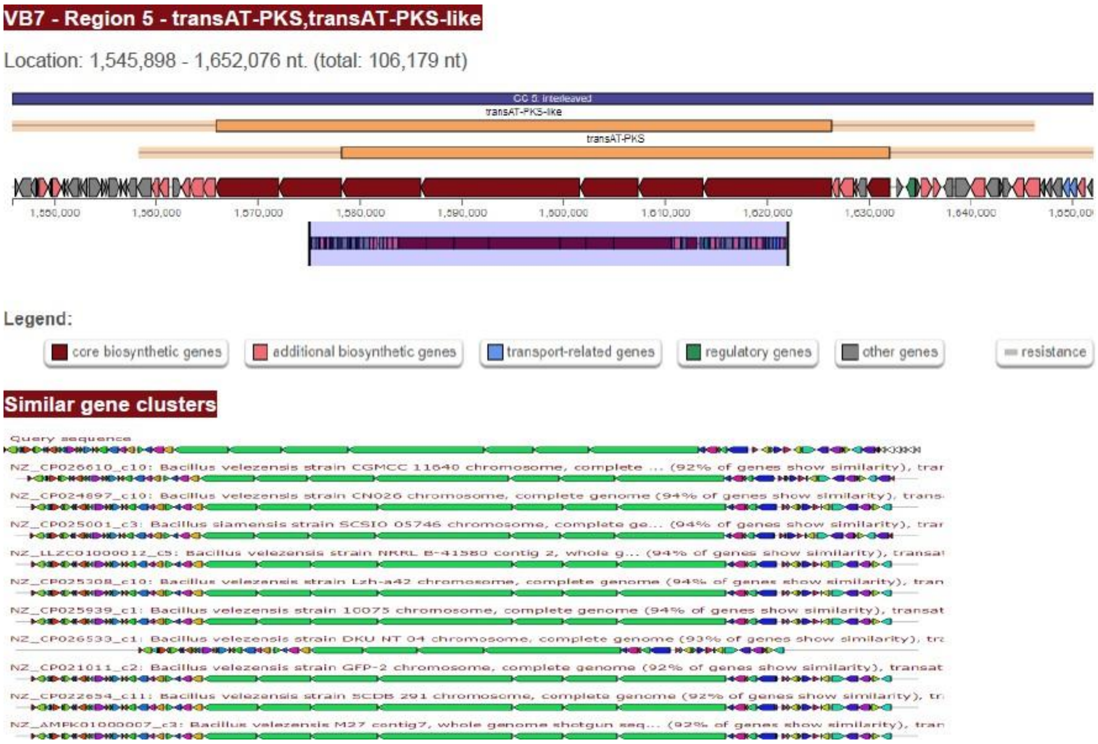

Figure S8 Region-6 coding for secondary metabolite, NRPS, bacteriocin in genome of *Bacillus velezensis* – VB7

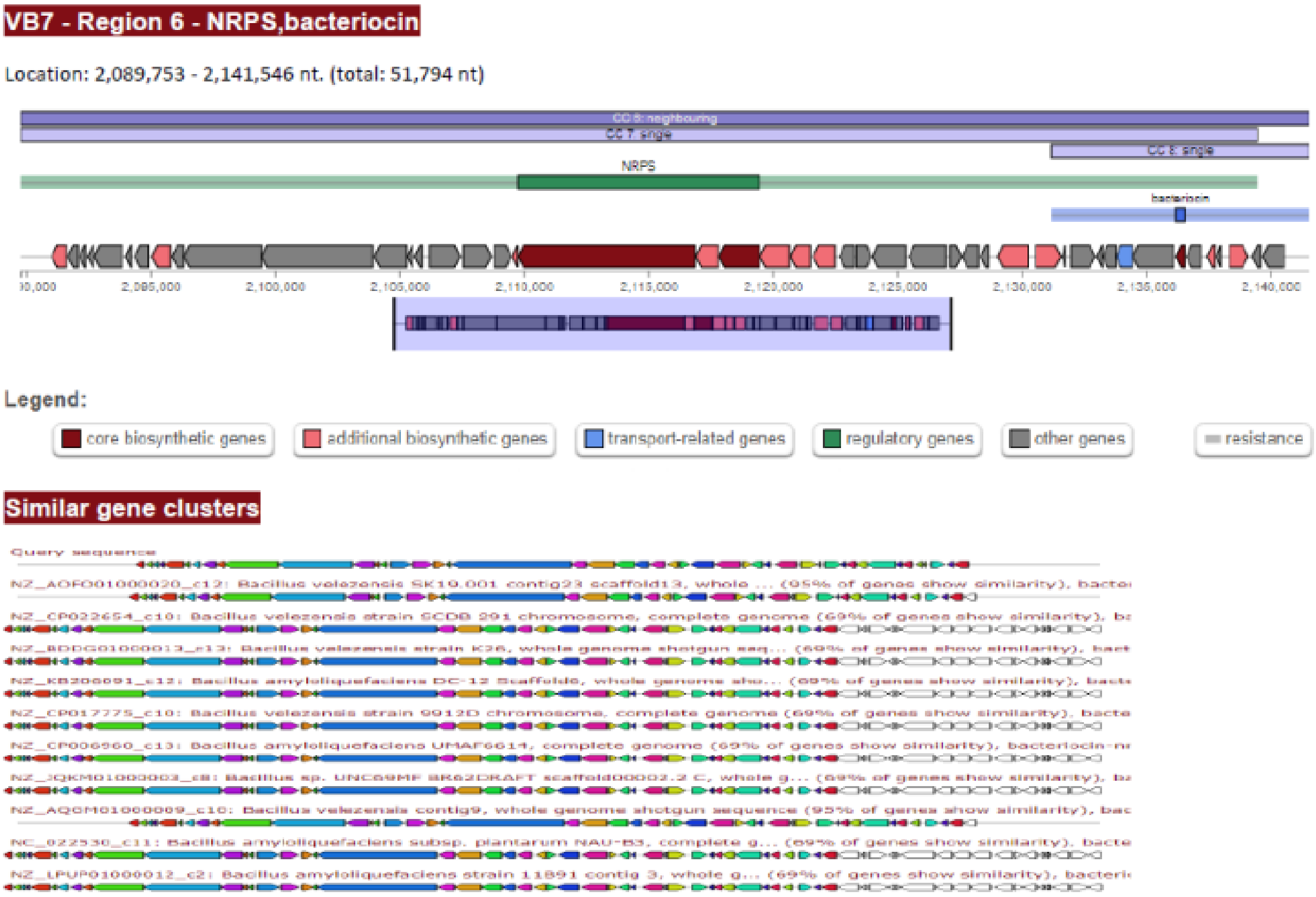

Figure S9 Region-7 coding for other secondary metabolite, bacilysin in genome of *B. velezensis* VB7

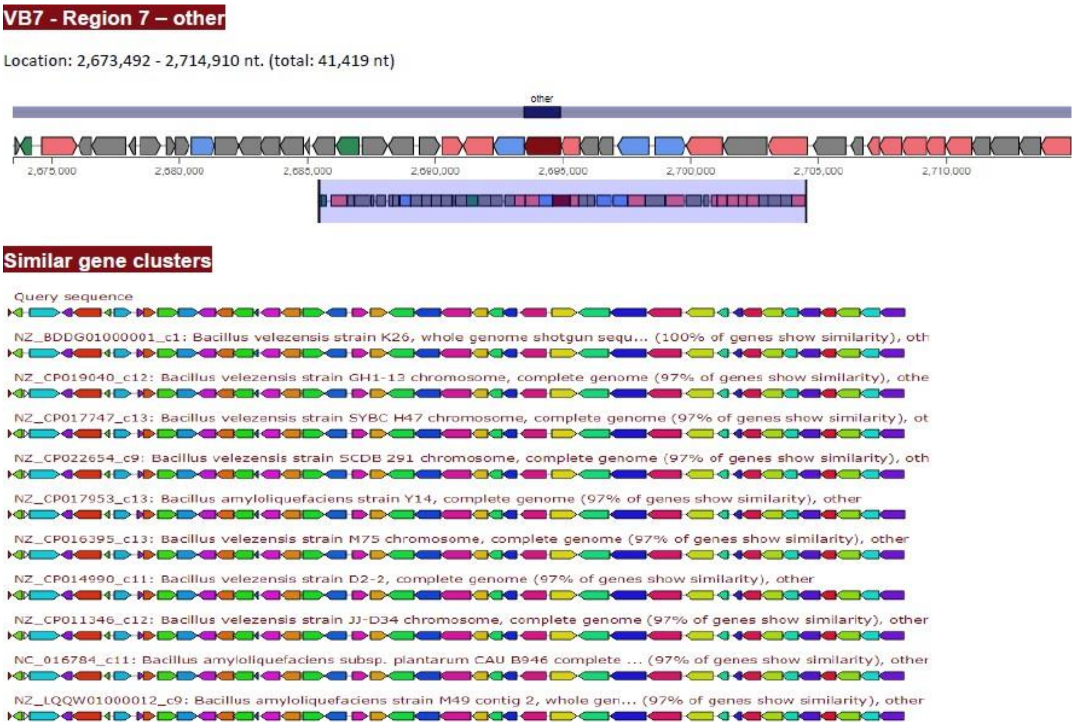

Figure S10 Region-8 coding for other secondary metabolite, lanthipeptide in genome of *B. velezensis* – VB7

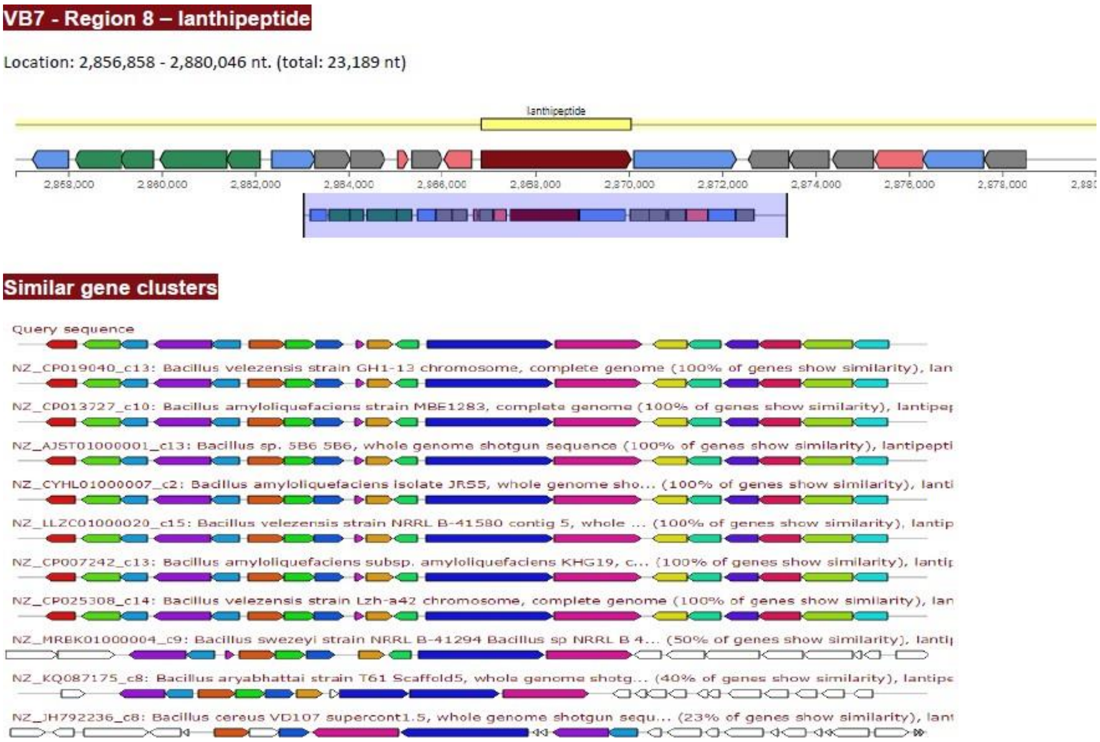

Figure S11. Antiviral efficacy of *Bacillus* spp., and chemical inducers on the suppression of tomato spotted wilt virus in cowpea local lesion host

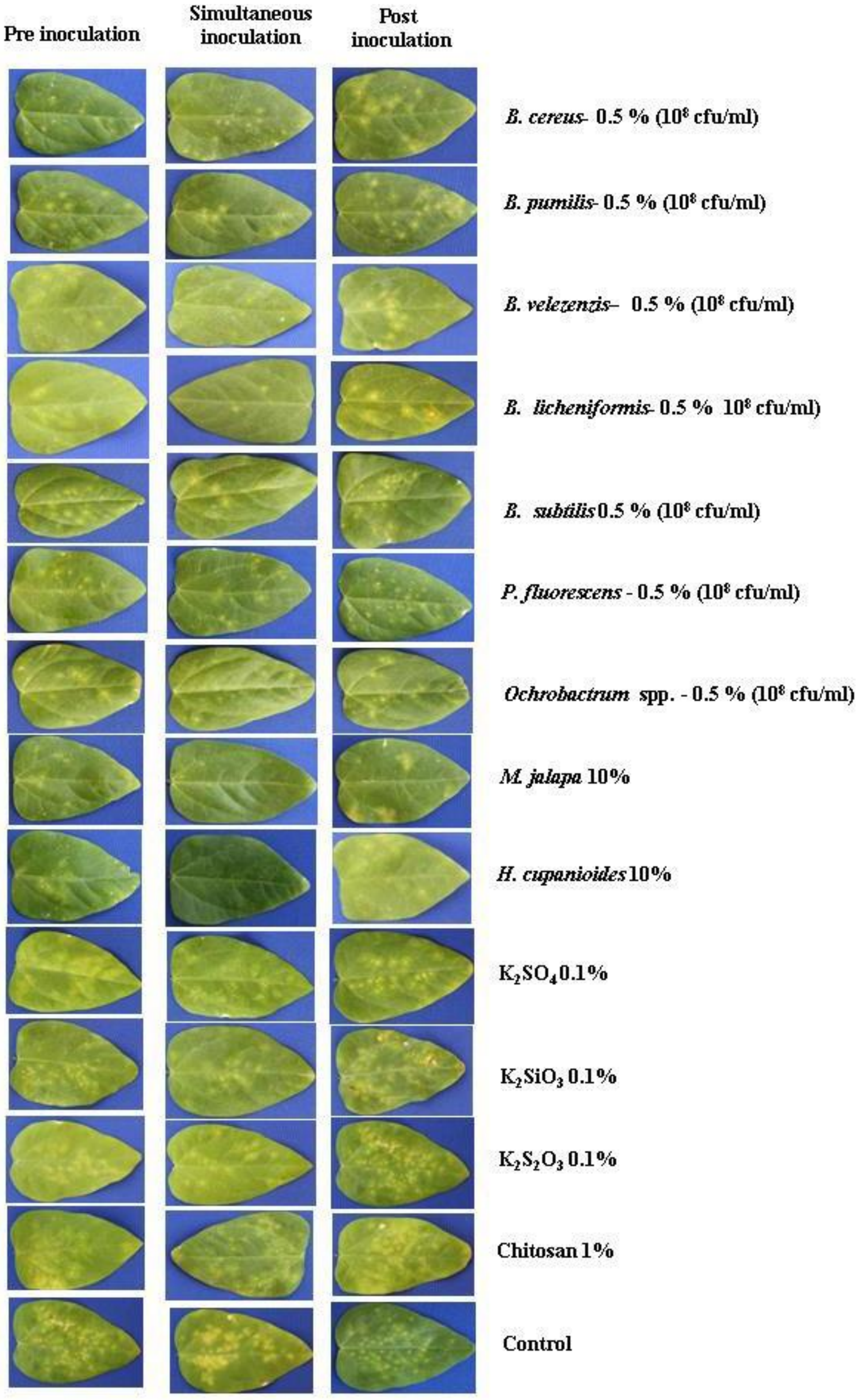

Figure S12 Evaluation of *B. amyloliquefaciens* VB7 and botanicals against TSWV on Chrysanthemum under field condition.

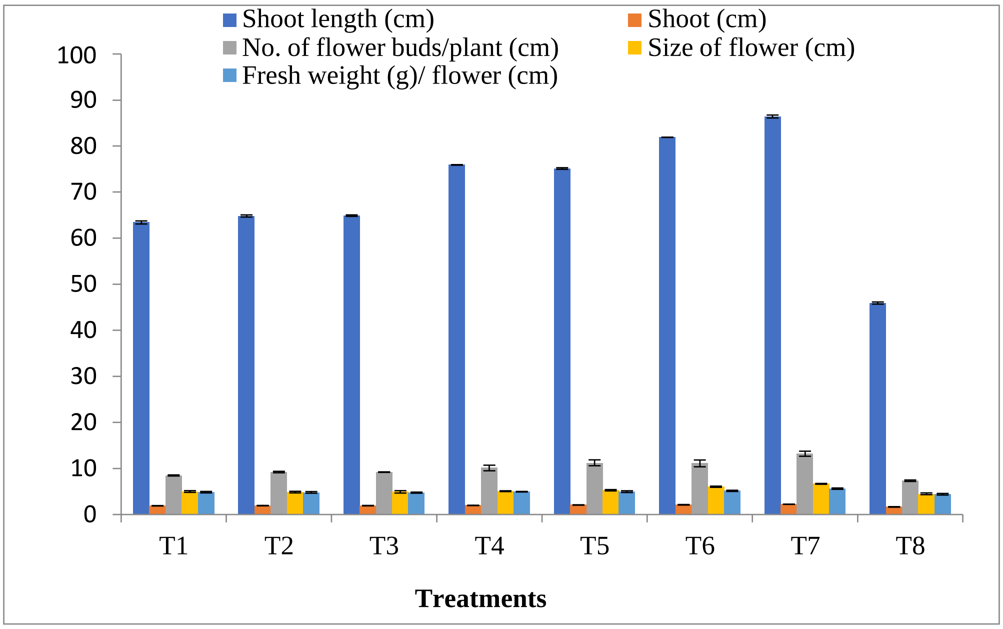

Note: T1 - *B. velezensis* VB7; T2 - *M. jalapa*; T3 - *H. cupanioides*; T4 - *B.velezensis* VB7 + *M. jalapa*; T5 - *B. velezensis* VB7 + *H. cupanioides*; T6 - *M. jalapa* + *H. cupanioides*; T7 - *B. velezensis* VB7 + *M. jalapa* + *H. cupanioides*; T8 - Untreated control

Figure S13 TSWV incidence and absorbance in chrysanthemum plants after the application of bio-control agents and botanicals under field condition

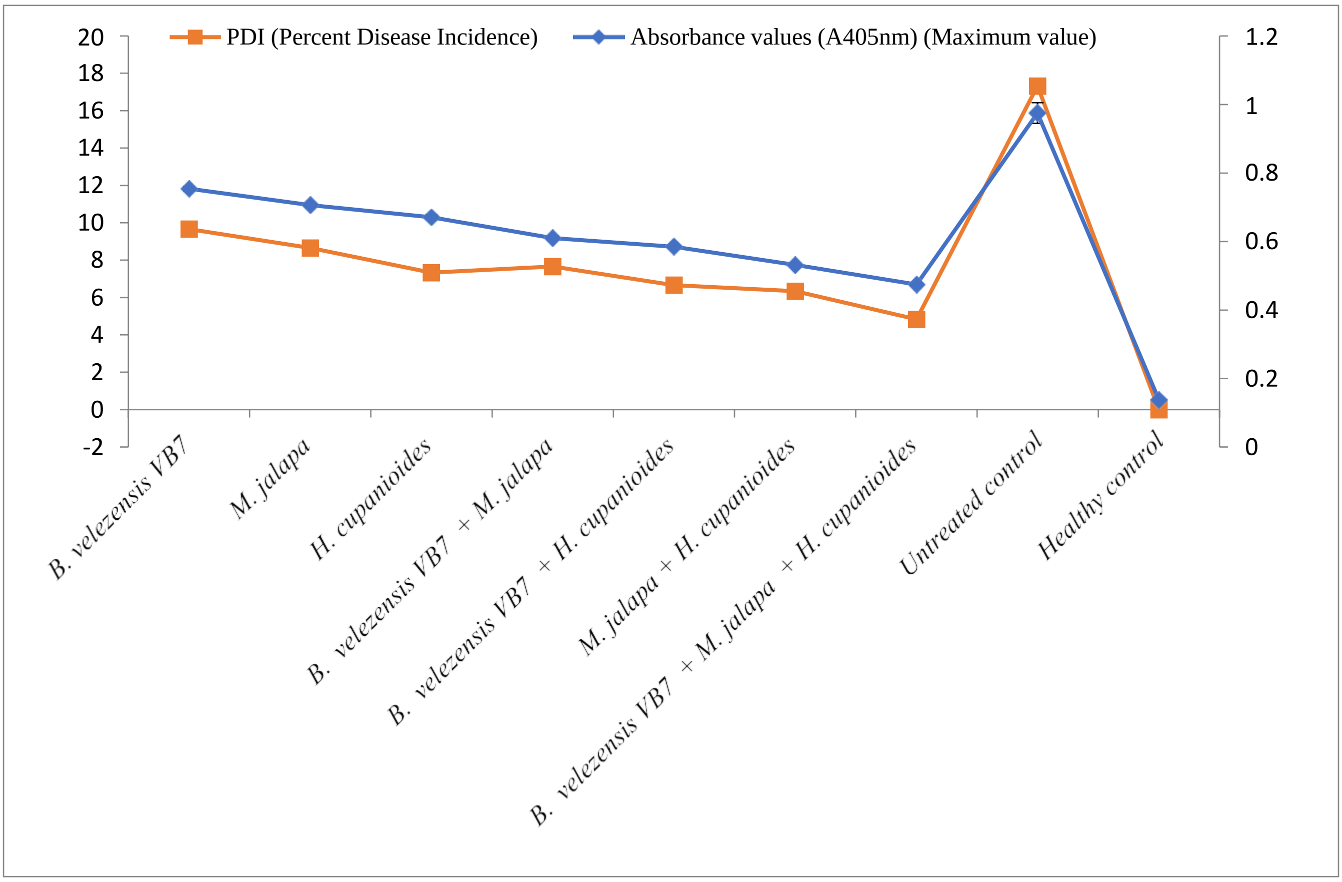

Figure S14. Antifungal activity of bacterial antagonists against *F. oxysporum* f.sp. *bense in vitro*

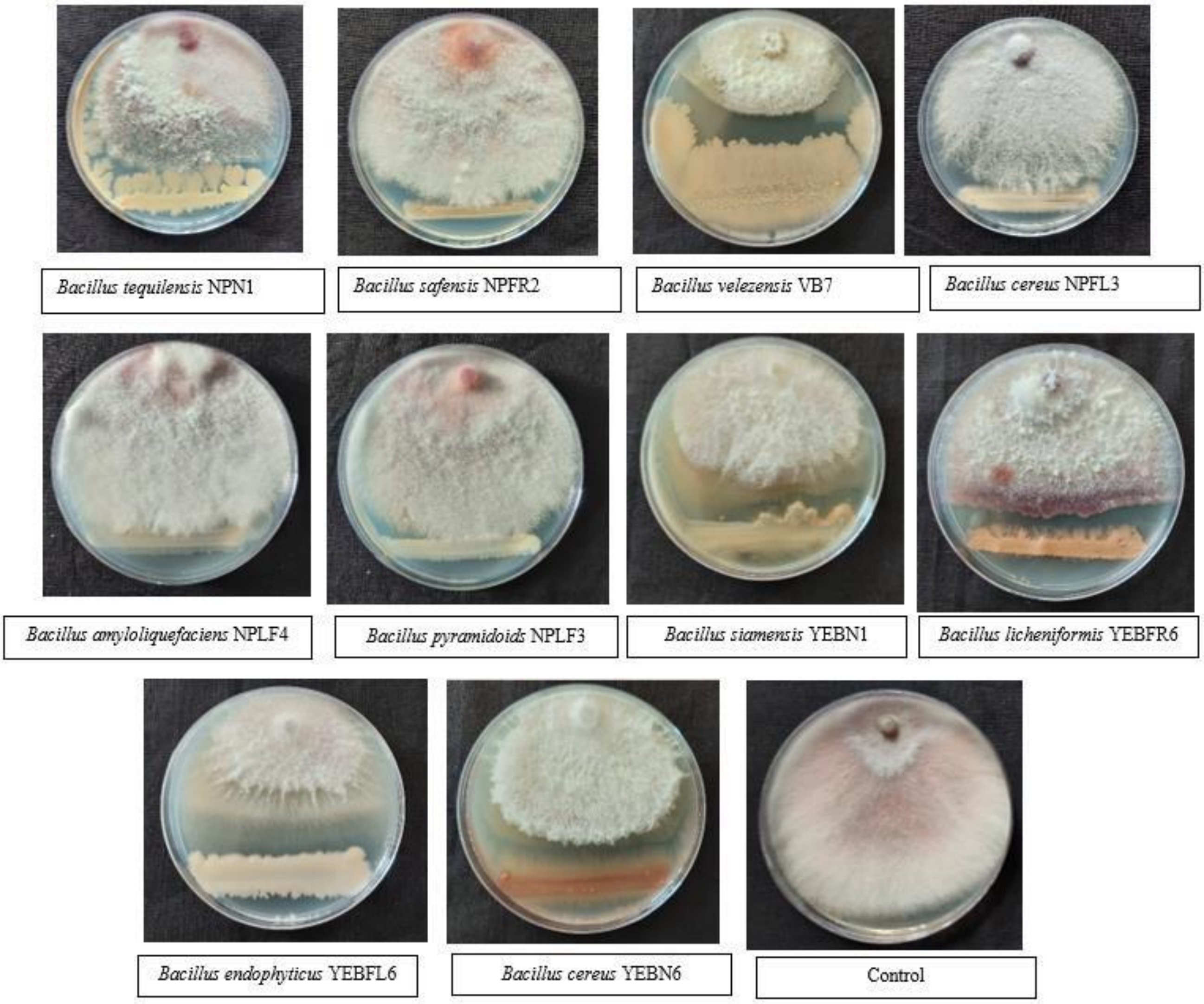

Figure S15 GCMS chromatogram of volatile and non-volatile organic compounds (VOC and NVOC) produced during the interaction of *Bacillus velezensis* + *Foc*

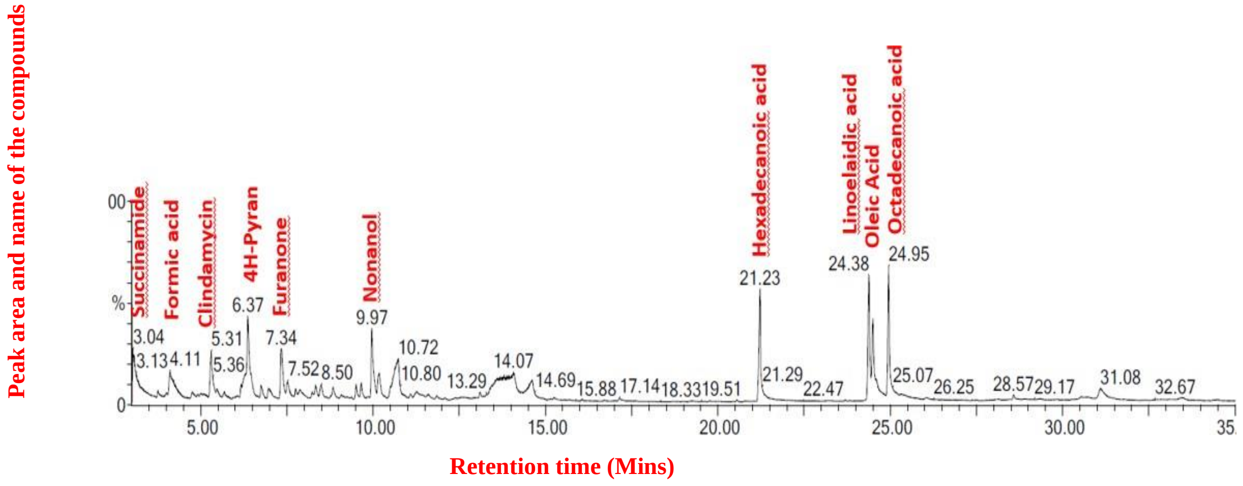

Figure S16 GCMS chromatogram of volatile and non-volatile organic compounds (VOC and NVOC) produced by *Bacillus velezensis* VB7.

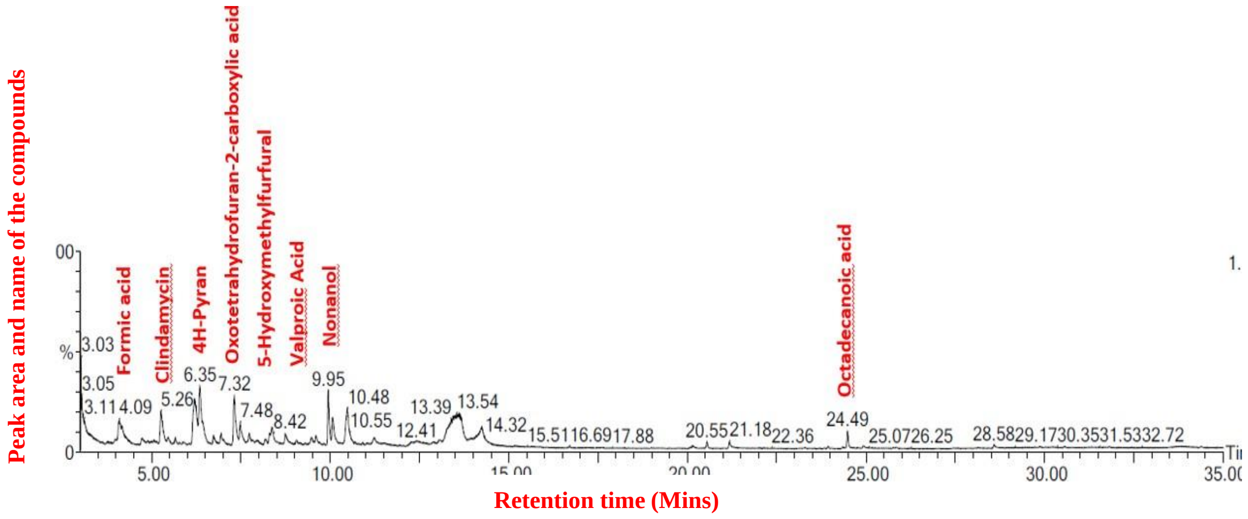

Table S1. Assembly details of the antagonistic bacterial isolate *B. velezensis* VB7 -CP047587

Contigs	1
GC Content	46.79
Plasmids	0
Contigs L50	1
Genome Length	3,021,183 bp
Contig N50	3,021,183
CDS	3,090
tRNA	85
rRNA	29
Repeat region	21
Partial CDS	0
Miscellaneous region	0
Hypothetical proteins	506
Proteins with functional assignments	2,584
Proteins with EC number assignments	822
Proteins with GO assignments	684
Proteins with Pathway assignments	607
Proteins with Subsystem assignments	858
Proteins with genus-specific family (Pfam) assignments	2,976
Proteins with cross-genus family (PGfam) assignments	2,976

Table S2. Annotation of virulence factor features in the genome of *B. velezensis* – VB7

Database	Gene	Product	Classification
Victors	codY	GTP-sensing transcriptional pleiotropic repressor CodY	-
Victors	clpX	ATP-dependent Clp protease ATP-binding subunit ClpX	-
PATRIC-VF	purB	Adenylosuccinate lyase (EC 4.3.2.2) @ SAICAR lyase (EC 4.3.2.2)	Virulence
PATRIC-VF	RsfA	Ribosomal silencing factor RsfA	Intracellular survival and replication
PATRIC-VF	purA	Adenylosuccinate synthetase (EC 6.3.4.4)	Intracellular survival and replication
VFDB	fliP	flagellar biosynthetic protein (FliP)	-

Table S3. Subsystem analysis of the genome *Bacillus velezensis* – VB7

**Super Class**	**No. of Subsystem**	**No. of families/ genes**
Metabolism	73	463
Cellular Processes	27	190
Stress response, defense, virulence	27	116
Protein processing	36	111
Energy	18	104
DNA processing	16	64
Membrane transport	14	63
RNA processing	10	36
Cell Envelope	4	15
Regulation and Cell signalling	3	11
Miscellaneous	3	7

Table S4. Details of unique genes in the genome of *B. velezensis* VB7 with their genomics position of coding sequence and protein product.

Genome start position	End position	Nucleotide Length	Strand (-/+)	Amino acid length	Protein
893183	893773	591	-	196	Phage-like element PBSX protein xkdA
893922	894260	339	-	112	Transcriptional regulator Xre, Cro/CI family
2296275	2297015	741	-	246	Carboxylesterase (EC 3.1.1.1)
2297134	2297364	231	-	76	Protein translocase membrane subunit SecG
2382458	2382598	141	-	46	UPF0311 protein BSU34410
2733044	2733793	750	-	249	FMN reductase (NADPH) (EC 1.5.1.38)
2733897	2735084	1188	-	395	Rod shape-determining protein RodA
2735286	2735744	459	-	152	Uncharacterized N-acetyl transferase YedL
2736342	2736710	369	-	122	Cytochrome aa3-600 menaquinol oxidase subunit IV
2736712	2737326	615	-	204	Cytochrome aa3-600 menaquinol oxidase subunit III
2737341	2739290	1950	-	649	Cytochrome aa3-600 menaquinol oxidase subunit I
2739318	2740283	966	-	321	Cytochrome aa3-600 menaquinol oxidase subunit II
2762549	2764060	1512	+	503	D-alanine--poly(phosphoribitol) ligase subunit 1 (EC 6.1.1.13)
2736038	2736298	261	+	86	Spore morphogenesis and germination protein YwcE
